# Gastric Splenosis Mimicking Gastrointestinal Stromal Tumor

**DOI:** 10.7759/cureus.12816

**Published:** 2021-01-20

**Authors:** Gowthami Kanagalingam, Vrinda Vyas, Vanessa Sostre, Muhammad Osman Arif

**Affiliations:** 1 Department of Internal Medicine, Upstate University Hospital, Syracuse, USA; 2 Department of Gastroenterology, Upstate University Hospital, Syracuse, USA

**Keywords:** splenic trauma, gastrointestinal stromal tumor (gist), endoscopic ultrasound (eus)

## Abstract

Translocation of splenic tissue in patients after traumatic spleen injury or splenectomy is called splenosis. Gastric splenosis is a rare presentation that can be mistaken for gastrointestinal stromal tumor (GIST). Patients are usually asymptomatic and do not require surgical intervention.

In this report, we present a case of a 68-year-old male patient with a previous history of surgical splenectomy after traumatic splenic rupture, who underwent routine upper endoscopy for the evaluation of dysphagia. An endoscopic exam of the stomach revealed an incidental finding of a submucosal gastric nodule. On endoscopic ultrasound exam, the lesion was found to be suggestive of GIST originating from layer 4. A core biopsy was obtained from the nodule, which was consistent with gastric splenosis.

The differentiation of gastric splenosis from other gastric lesions such as GIST is important since asymptomatic patients with gastric splenosis do not need to undergo surveillance or surgical resection. It should be suspected especially in patients with a history of splenectomy or splenic rupture. Endoscopic ultrasound (EUS)-guided core biopsy can help confirm the diagnosis and differentiate the condition from GIST.

## Introduction

Splenosis is the translocation of splenic tissue, mostly caused by a traumatic injury to the spleen or iatrogenically after surgery [1]. It is usually seen in the abdominal and pelvic cavity, with gastric splenosis being a rare entity [2]. Gastric splenosis is frequently mistaken for gastrointestinal stromal tumor (GIST). Confirming the diagnosis of gastric splenosis and differentiating it from GIST is important to avoid unnecessary endoscopic surveillance and surgical treatment [3,4].

## Case presentation

A 68-year-old male patient presented to the emergency room for the treatment of esophageal food impaction. His past surgical history was significant for colectomy after intestinal rupture and splenectomy due to traumatic splenic rupture. Endoscopic evaluation incidentally showed a medium-sized submucosal papule in the gastric fundus. Superficial biopsy results from the area showed fundic type gastric mucosa. 

The patient underwent endoscopic ultrasound (EUS) to follow up on the gastric nodule. It revealed a single hypoechoic subepithelial papule in the stomach originating from within the muscularis propria (layer 4), with 9-mm thickness and 4-mm diameter, raising suspicion about benign stromal cell neoplasm (GIST). It was thought to be benign, given the small size, and hence, no tissue biopsy was obtained at the time. A repeat EUS six months later did not show any change in the size of the lesion; therefore, follow-up was recommended in two years.

**Figure 1:**
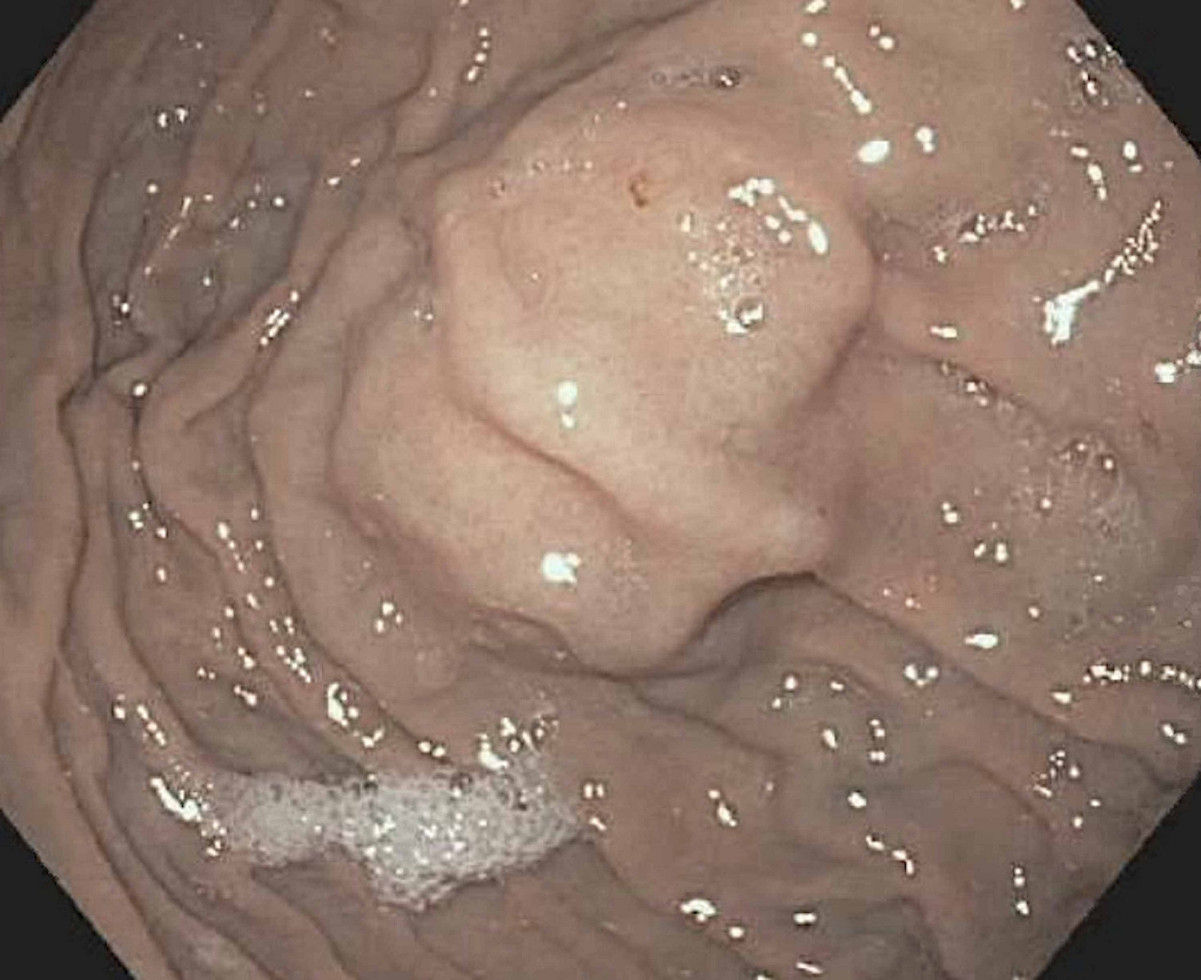
Submucosal papule in the fundus of the stomach

**Figure 2:**
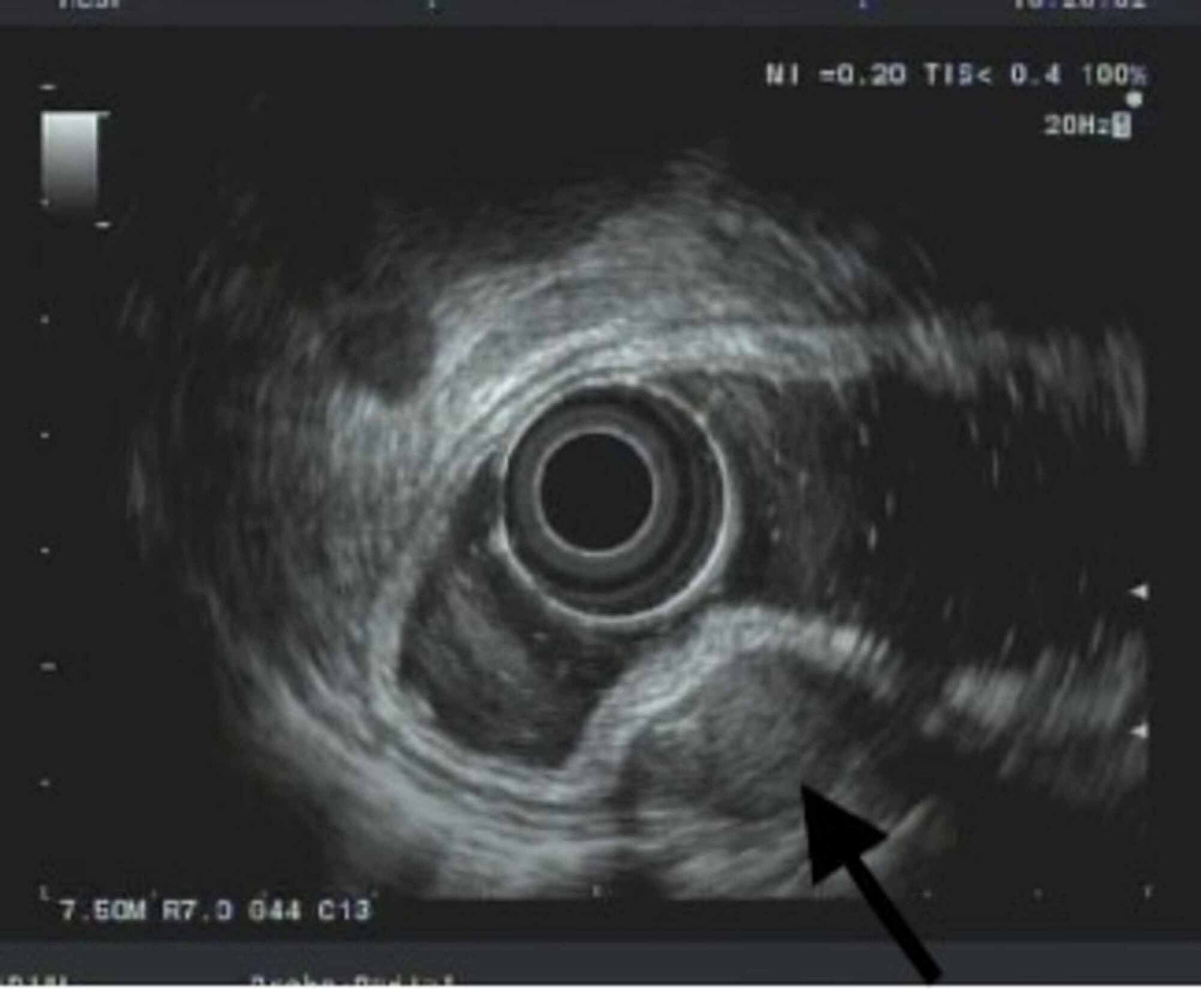
Submucosal papule that appears to be originating from within muscularis propria, layer 4

**Figure 3:**
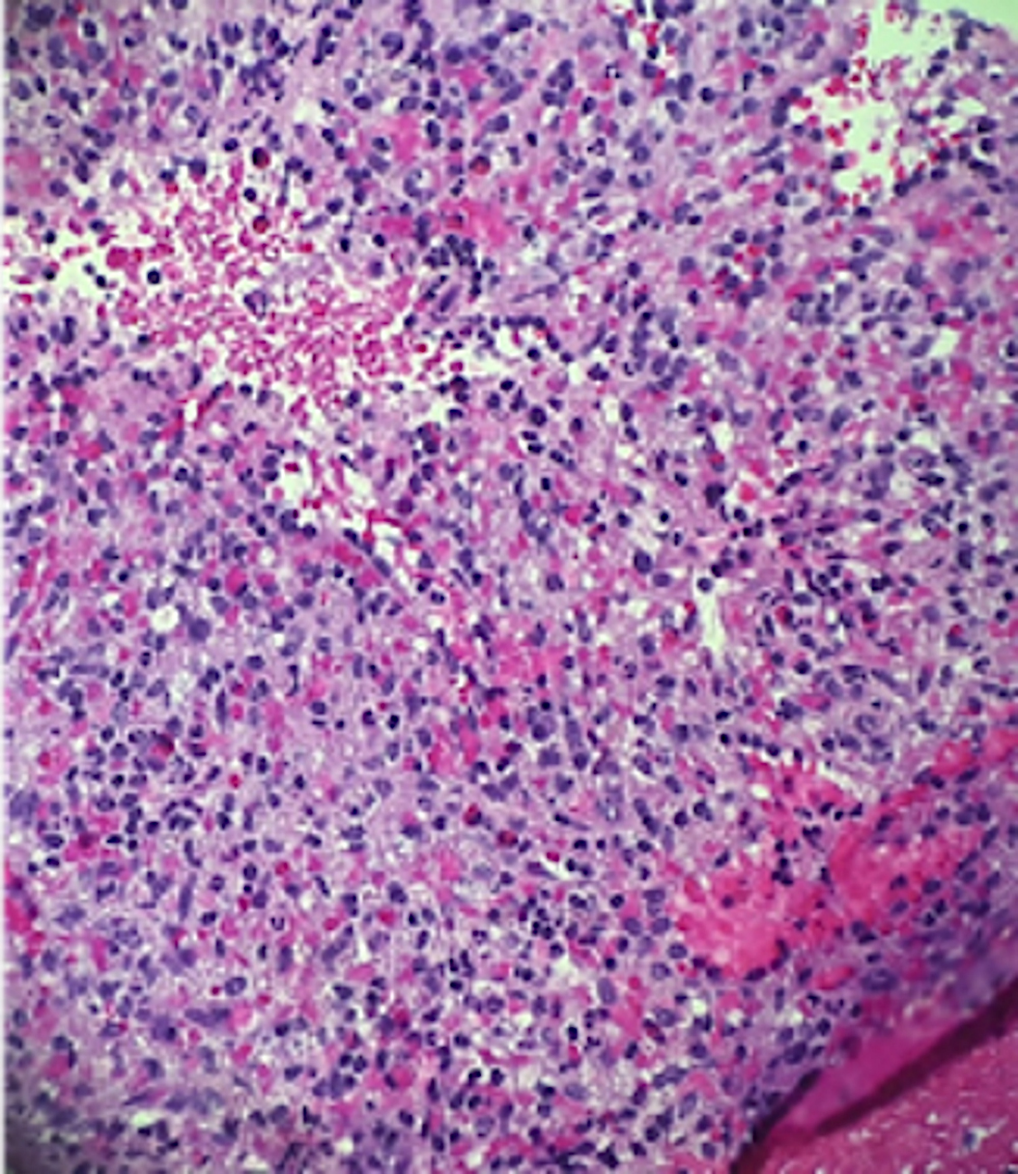
Core biopsy of the lesion showing multiple soft tan-white tissue cores composed of blood and eosinophilic stroma with eosinophils, macrophages, plasma cells, and neutrophils consistent with gastric splenosis

Differential diagnosis at that time included leiomyoma or GIST. The patient did not exhibit any symptoms related to the gastric nodule during the follow-up period and specifically denied abdominal pain, nausea, vomiting, or blood in the stool. EUS was repeated at a two-year interval, which showed an intramural lesion in the fundus of the stomach originating from layer 4, consistent with the previous exam (Figure 1, Figure 2). EUS-guided core biopsy was obtained. Immunohistochemistry revealed diffuse expression of CD8, which is a specific marker for littoral cells, a distinctive type of endothelial cells unique to splenic tissue; thus, the tissue biopsy was reported as gastric fundal splenosis (Figure 3). No further surveillance exams were recommended.

## Discussion

The term splenosis represents the auto-transplantation of splenic tissue [1]. It was initially described in 1937 [2]. It is mostly seen in the abdominal and pelvic cavity including the greater omentum, serosa of the intestines, mesentery, parietal peritoneum, and diaphragm [3,5]. Other sites such as intrathoracic, gastric, intrahepatic, kidney, brain, and lungs have also been described [2]. Gastric splenosis is rare with less than 20 case reports in the literature until 2018 to the best of our knowledge [2]. The exact incidence of the condition is unknown as it is often an incidental finding [6].

The cause of splenosis is thought to be trauma to the spleen in 70% or iatrogenic in 30% of cases [2]. Hypotheses for the possible pathogenesis of gastric splenosis include either direct translocation during splenic surgery or intra-organ seeding via vasculature [2]. Patients with gastric splenosis are mostly asymptomatic [3]. Symptomatic patients usually present with abdominal pain or with torsion, spontaneous rupture, or gastrointestinal bleeding [6,7].

Gastric splenosis is definitively diagnosed with tissue pathology, which may not consist of all types of cells of normal spleen tissue. On histology, splenosis presents as tubercles, which consist mostly of reticular cells and fibrous tissue. Splenic tissue in splenosis usually lacks follicle, germinal center, and columnar structure [6]. Other useful diagnostic tests include Tc-99m heat-denatured RBC spleen scintigraphy and iron oxide-enhanced MRI [2]. It can be easily mistaken for GIST based on EUS examination alone.

Treatment for gastric splenosis is only required for patients with symptoms such as abdominal pain or gastrointestinal bleeding. Depending on the size and location, surgical resection via endoscopy or laparoscopy may be indicated [2].

This article was presented as an abstract at the American College of Gastroenterology (ACG) Conference 2020 on October 26.

## Conclusions

Gastric splenosis should be part of the differential diagnosis in patients with incidental findings of submucosal gastric lesion and prior history of splenectomy. EUS-guided core biopsy can help obtain a definitive diagnosis, which may alter the management plan since asymptomatic patients do not need surgical resection or surveillance.
